# The role of CXCL family members in different diseases

**DOI:** 10.1038/s41420-023-01524-9

**Published:** 2023-07-01

**Authors:** Chenjia Zhou, Ying Gao, Peilun Ding, Tao Wu, Guang Ji

**Affiliations:** 1 Institute of Digestive Disease, Longhua Hospital, Shanghai University of Traditional Chinese Medicine, 200032 Shanghai, China; 2 Institute of Interdisciplinary Integrative Medicine Research, Shanghai University of Traditional Chinese Medicine, 201203 Shanghai, China

**Keywords:** Cytokines, Inflammation

## Abstract

Chemokines are a large family mediating a lot of biological behaviors including chemotaxis, tumor growth, angiogenesis and so on. As one member of this family, CXC subfamily possesses the same ability. CXC chemokines can recruit and migrate different categories of immune cells, regulate tumor’s pathological behaviors like proliferation, invasion and metastasis, activate angiogenesis, etc. Due to these characteristics, CXCL subfamily is extensively and closely associated with tumors and inflammatory diseases. As studies are becoming more and more intensive, CXCLs’ concrete roles are better described, and CXCLs’ therapeutic applications including biomarkers and targets are also deeply explained. In this review, the role of CXCL family members in various diseases is summarized.

## FACTS

1. Chemokines regulate the biological behaviors of leukocytes and tumor cells, and play an essential role in the development of carcinomas and inflammation. CXCLs, one subfamily of chemokines, are irreplaceable in these diseases through their relevant functions including the recruitment of immune cells and engagement in the pathological process of neoplasm.
2. Researches assoiciated with CXCLs and their relevant diseases mainly focus on cancers and inflammatory diseases. It has been proved that CXCLs are closely involved in various cancers, such as breast cancer, lung cancer, gastric cancer, hepatoma, colorectal cancer, ovarian cancer, pancreatic cancer, prostate cancer, melanoma, etc. CXCLs also closely participate in inflammatory diseases appearing in different organs and systems, such as arthritis, systemic lupus erythematosus, chronic obstructive pulmonary disease, hepatitis, etc.
3. Recently it has been confirmed that CXCLs control inflammation and immune responses through the migration of leukocytes including neutrophils, monocytes, macrophages, etc. They also regulate tumor cells proliferation, invasion, metastasis, and angiogenesis by activating different signaling pathways like STAT3 and NF-κB, thus accelerating the development of cancers. However, the concrete mechanisms how CXCLs exert their functions in these diseases are still not completely clarified.
4. Many researches have considered CXCLs as potential biomarkers, targets and indicators in cancers, inflammatory diseases, and metabolic diseases. The potential value of CXCLs has been extensively recognized in diagnosis, prognosis and therapy of these diseases, whereas their clinical translation is still not fully explored.

## Open questions

1. How to concretely participate in clinical diagnosis, prognosis and therapy of CXCLs associated diseases through utilizing CXCLs?
2. Can CXCLs be used in anti-tumor immunotherapy through their role in recruitment of immune cells?
3. Can CXCLs be used as a practical target to help wound healing via promoting angiogenesis in clinical treatment?

## Introduction

Chemokines are small-molecule secreted proteins whose basic function is stimulating the migrations of cells, especially leukocytes. Its functions are implemented via the interaction with G-protein coupled receptors on the cell surface [1]. Those chemokines are involved in the recruitment of leukocytes and control of the tumor microenvironment, thus regulating the tumor cell proliferation, metastasis, invasion, angiogenesis, and therapeutical resistance [2]. Chemokines has been served as a key point of studies for lots of complex diseases due to their high engagement in life activities. Presently, they have been found in a variety of biological processes, including tumor growth, wound healing, immune system homeostasis, and embryonic development. Thus, abnormal chemokines’ expression usually implies an abnormality in the biological processes in which they participate, reflecting the development of relevant diseases, such as carcinomas, inflammatory disorders and metabolic diseases [3].

According to the differences in conserved cysteine motif which is close to N-terminus, chemokines are classified into four groups: CCL, CXCL, XCL and CX_3_CL families [4]. CXCL family is one of the main chemokine families. The small proteins in this family are secreted by tumor cells, leukocytes, fibroblasts, endothelial cells, and epithelial cells [5]. As one member of chemokine family, the influence of CXCL family expression in chemokine-linked diseases cannot be ignored.

Increasingly, CXCL chemokines have been found to make a notable effect on the progression and growth of neoplasm, such as breast cancers, kidney renal clear cell carcinoma, hepatocellular carcinoma (HCC), ovarian cancer and so on. They have great potential to be therapeutic targets for these diseases in the future. Similarly, CXCL family play a complicated role in the occurrence of inflammatory reaction, which provide a strong support for treatments of inflammation-related disorders such as chronic obstructive pulmonary disease (COPD), arthritis and even some autoimmune diseases [6]. Meanwhile, CXCLs’ explorations on metabolism have also begun with some progress in recent years. Therefore, researches focusing on the value of CXCL family about therapy strategy of relevant diseases are remarkably increasing. The intensive discoveries of CXCLs in terms of expressions or mechanisms of action bring deeper insight into their value of diagnosis, prognosis and therapy.

This paper is aim to review the CXCL family and elaborate the updated relative researches of the role of CXCL family members in different diseases, providing a foundation for further research about pre-clinical and clinical applications.

## The structure and function of CXCL family

In four highly conservative cysteines, some chemokines have one uncertain amino acid between two cysteines and form C-X-C motif, which is the characteristic of CXC chemokines. According to the existence of ELR motif (Glu-Leu-Arg) located at N-terminus, CXC chemokines can be divided into ELR^+^ or ELR^-^ CXC chemokines, which means angiogenesis activators or inhibitors. ELR^+^ CXC chemokines include CXCL1, CXCL2, CXCL3, CXCL5, CXCL6, CXCL7, CXCL8 and CXCL17 while ELR^-^ CXC chemokines include CXCL4, CXCL9, CXCL10, CXCL11, CXCL12, CXCL13, CXCL14 and CXCL16 [4, 7]. ELR^+^ members are combined with CXCR1 or CXCR2, while ELR^-^ members are combined with CXCR3, CXCR4, CXCR5, CXCR6, and CXCR7. All of them usually activate MAPK, PI3K/AKT, STAT3, NF-κB, Ras, TGF-β and β-catenin signaling pathways for the development of tumor diseases [7].

CXCL1, known as growth related oncogene, can be expressed in many immune cells and neurons. The main function of CXCL1 is recruitment of immunocytes, especially neutrophils [8]. It can promote proinflammatory reaction, immune regulation and angiogenesis. CXCL2 is also a growth related oncogene like CXCL1, produced by activated monocytes and macrophages. It has chemotaxis of neutrophils. Like CXCL1 and CXCL2, CXCL3 is also a chemotactic factor of neutrophils. CXCL4, called as platelet factor 4, is a small protein released from activated platelets. It exerts many functions like hemostasis and stimulation of immune cells at site of tumor [9]. CXCL5, known as epithelial activating neutrophil protein, plays a role in immune system and inflammation. CXCL6, also known as granulocyte chemotactic protein 2, is a chemoattractant for neutrophils through receptors CXCR1 and CXCR2. It is closely related to cell permeability, apoptosis and proliferation [10]. CXCL7, also called neutrophil activating peptide 2, is secreted by monocytes and macrophages to promote tumor progression [11, 12]. CXCL8, secreted by macrophages and epithelial cells, is an active angiogenic factor and proinflammatory factor, taking part in the cancer development, including change of tumor microenvironment, tumor migration, tumor proliferation and so on. It possesses dual pro-tumorigenic effect [13]. CXCL9, CXCL10 and CXCL11 has the same receptor CXCR3. They are secreted by leukocytes, macrophages, tumor cells and fibroblasts. They can be induced by IFN to recruit Th cells, T cells, natural killer cells to perform antiinfection and antitumor role [14]. CXCL12, known as stem cell-derived factor 1, has angiogenesis property and regulate tumor progression. CXCL13, expressed by stromal cells and macrophages, is an effective B cell chemoattractant [15]. It also plays a role in cancer development. CXCL14 is a highly conserved chemokine, performing a crucial role in infiltration of immune cells, cell mobilization, and maturation of dendritic cells. CXCL14 derived from different cells affects tumor progression in different ways. CXCL14 from fibroblast promotes tumor development. CXCL14 from epithelial cells, conversely inhibits tumor development [16]. CXCL16 is a chemokine expressed in liver, lung, kidney, etc. It recruits natural killer cells to defend against tumor mainly [17]. CXCL17 can be produced by airway epithelium, playing positive and pathological role respectively in different cancers. It is chemoattractant of monocytes and macrophages. It also participate in recruiting myeloid cells, inhibiting pathogenic microorganisms and tumor angiogenesis [18].

As one of the subfamilies among chemokines, CXCL family can not only regulate the migration of leukocytes, but also control the growth and development of tumor. Based on lots of researches, it has been proved to involve in the regulation of immune cell activity, the proliferation, invasion and metastasis of tumor cells, neoplastic microvascular formation, transformation of tumor cells. It also changes the angiogenic environment, promotes the growth of local tumor cells, enters the circulatory system through the invasion of extracellular matrix (ECM) and vascular basement membrane, and metastasizes to distant organs. Some CXCLs can migrate stem cells to repair wounds or injury. These actions of CXCL are closely related to inflammatory reactions, immune diseases, cancers and so on [19].

## CXCL family and diseases

### Tumor and cancer

#### Lung cancer

Lung cancer, which is closely concerned with CXCL family, is the most common cancer and the predominant cause of neoplasm-related deaths all over the world. For example, several studies had reported that CXCL1, CXCL2 and CXCL8 are considered as participants and promoters in the occurrence of lung cancer by various mechanism, including recruiting neoplasm-associated neutrophils, participating in anlotinib resistance and influencing the proliferation of tumor cells and angiogenesis [20, 21]. Nese Unver et al. found that CXCL1, CXCL5, CXCL7 and CXCL8 were the main angiogenic CXC chemokines in non small cell lung cancer (NSCLC), which can make immune cells including neutrophils and macrophages tumor-infiltrating to support the development of tumor [22]. Thus, it indicated that serum chemotatic factors like CXCL8 have the possibility of differential diagnosis of lung cancer such as NSCLC [23]. CXCLs also partipate in tumor microenvironment. Wang et al. concluded that CXCL12-CXCR4 axis regulated epithelial-mesenchymal transition (EMT) in tumors, induced angiogenesis, and promoted neoplasm metastasis and development [24]. Wu et al. discovered that transmembrane glycoprotein CD248-expessed cancer associated fibroblasts can develop NSCLC via the secretion of CXCL12 and induction of M2 polarization of macrophages [25]. Chao et al. found that CXCL13-CXCR5 axis promoted cell motility in lung cancer cells which were mediated by VCAM-1 expression. Meanwhile, they also indicated that PLCβ, PKCα and c-Src signaling pathways also participated in CXCL13-promoted cell migration and VCAM-1 expression in lung cancer cells [26].

#### Gastric cancer

Gastric cancer (GC) also shows the actions of CXCL family. Gastric cancer is one kind of solid tumor that contains tumor cells and diverse stromal cells. CXC chemokines and their receptors participate in the growth and development of GC. Chen et al. studied their roles in GC. It was found that the concentrations of CXCL1, CXCL2, CXCL4, CXCL5, CXCL7, CXCL8, CXCL9, CXCL10, CXCL12, CXCL13 and CXCL14 in tumor drainage blood and peripheral blood were considerably higher than those in patients without recurrence, as well as the down-regulation of CXCL factors’ expression inhibited cell lines’ ability to migrate. All of these indicated the important role of CXCL family in gastric cancer [27]. Besides, a research of CXCL family related to Epstein-Barr virus (EBV) associated gastric cancer has elucidated that the expression differences of CXCL family members were closely related to the progression of EBV-associated GC, including the relationship with clinical stage, virus infection, prognosis, etc. It was reported that changes in CXCL9, CXCL10, CXCL11 and CXCL17 mRNA expression had potential prognostic values in patients with EBV-associated GC [28]. Moreover, Zhou et al. had discovered that macrophages, which were induced by the secretion of TNF-α from GC cells released CXCL1 and CXCL5. These two chemokines can accelerate migration and of GC cells by activating CXCR2-STAT3 feed forward loop. According to the results above, they suggested that CXCR2, the receptor of these two CXC chemokines, might be an effective therapeutic target to inhibit the progression and metastasis of GC [29]. Based on previous studies, it can be deduced that the vast majority of CXC chemokines are overexpressed in GC so that they and their receptors can be considered as targets and biomarkers of GC. Their prognosis values for GC are fully reflected [27, 28].

#### Colorectal cancer

Colorectal cancer (CRC) is concerned with CXCL family too. Since CRC is the leading diagnosed cancer in the world, it is necessary to find more useful biomarkers to diagnose this disease. Regarding the pathology of CRC, Luo et al. indicated that the high-level expression of CXCL family can lead to lymph node metastasis and a higher tumor-node-metastasis stage of CRC in CRC patients. In their study, worse tumor features and poor overall survival in patients with CRC were influenced by CXCL1, CXCL2, CXCL8 and CXCL13, which contributed to CRC treatments [30]. The relationship of CXCL1 and CRC has been studied. Zhuo et al. had explored the mechanism of actions for CXCL1 affecting the development of CRC, and they found that through the stimulation of NF-κB/P300 signaling pathway CXCL1 facilitated tumor cell proliferation and migration as well as promoted angiogenesis [31]. CXCL2 is no exception. CXCL2 has been elucidated that it has important biological effect on colon cancer, including inducing dose-dependent proliferation and migration. It can also regulate angiogenesis and peritoneal metastasis through its interactions with CXCR2 [32]. Besides, several evidences indicated that CXCL11, which regulated the chemotaxis of cells and induced infiltration of tumor-associated macrophages, was also one of the key chemokines providing a link between inflammation and CRC [33]. Other studies also suggested that CXCL12-CXCR4/7 axis played a significant part in the tumorigenesis, invasion, metastasis and angiogenesis of CRC so that it was considered to be a possible therapeutic target [34]. For example, Wang et al. indicated that exosome-encapsulted miRNAs were able to strengthern M2 polarization of macrophages via induction of CXCL12-CXCR4 axis, leading to liver metastasis of colorectal cancer cells [35]. In addition, it was reported by several researches that CXCL17 was involved in the pathogenesis of CRC, too [18].

#### Breast cancer

Breast cancer (BC) is the most frequent tumor affecting women, whereas CXCLs have the potential role in the treatment of BC according to the recent researches. For instance, Mishra et al. explained the dual responsibility of promoting tumor formation and suppression of tumors performed by CXCL8-CXCR1/2 axis in BC, suggesting the value of the targeted regulation of this axis for the prognosis, analysis and therapy of BC [36]. Also, since CXCL8 played a significant role in stimulating tumor angiogenesis and promoting migration and invasion of tumor cells, Motyka et al. combined with CA15-3 to analyze the diagnostic utility of CXCL8 in BC and demonstrated that CXCL8 could be used as an extra diagnostic marker that had positive effect on the diagnostic utility of marker CA 15-3 [37]. Another study suggested that the CXCL13-CXCR5 axis was closely related to the growth of BC and was regarded as a good prognostic marker of BC [38]. Besides, the role of CXCL17-CXCR8 axis in BC was showed by Heshemi et al. which was promoting the neoplasm cell proliferation and migration [18]. Hsu et al. demonstrated that CXCL17 secreted from breast cancer cells can make myeloid-derived suppressor cells accumulated in the lung, ultimately inducing angiogenesis and lung metastasis of breast cancer cells [39]. Furthermore, Hozhabri et al. had reported that CXCL4, CXCL9, CXCL12, and CXCL14 were also significantly correlated with the overall survival (OS) or clinical outcomes of BC [40]. All of these can provide a direction to find the clinical practice of CXCLs in BC.

#### Pancreatic cancer

Pancreatic cancer (PC) is also one of the most commonly diagnosed cancers [41]. Recent researches indicated that most of CXCLs were markedly upregulated in PC. It was showed that the levels of CXCL1, CXCL3, CXCL5, and CXCL8 expression were notably associated with clinical stage of PC. Meanwhile, CXCL5 expression was significantly related to tumor progression and survival time [42, 43]. Ala Litman et al. illustrated the significance of the CXCL8-CXCR2 axis in PC, which indicated that the serum CXCL8 was superior to CXCR2, C-reactive protein, classic tumor markers CA 19-9, and CEA when used as a diagnostic and predictive marker [44]. What’s more, Huang et al. explored the expression level and biological functions of CXCLs in pancreatic adenocarcinoma, including tumor angiogenesis, overall survival, immune dysfunction and so on. Their efforts provided further progression of the prognosis and immune treatment of PC [45]. Thus, CXCL family made a certain effect on the occurrence and development of PC.

#### Liver cancer

Liver cancer can be divided into two parts: primary liver cancer and secondary liver cancer. The most frequent primary liver is HCC, which is closely related to the role of CXCL family [46]. For instance, CXCL5 was considered to have the ability to promote HCC cell proliferation, migration and invasion via activating PI3K and ERK1/2 pathways and neutrophil infiltration [46, 47]. Conversely, CXCL2 overexpression takes part in the inhibition of ERK1/2 signaling pathways and promotes apoptosis, therefore suppressing HCC development. CXCL2’s role as a tumor suppressor was first determined in the study carried out by Ding et al. [48]. CXCL8 is also involved in tumor development. One study has demonstrated that the interaction between HCC cells and macrophages promoted tumor cell proliferation, migration and metastasis through the upregulation of CXCL8/miR-17 cluster [49]. Moreover, another study had revealed that CXC chemokines can regulate the interactions between immune cells and HCC cells in a tumor microenvironment. They discovered the relationship between CXCL mRNA expression and overall survival, as well as revealed six types of immune cells (including macrophages, dendritic cells, B cells and so on), the infiltration of which were affected by these chemokines [50]. Lin et al. explained the extensive relationship between CXCL2, CXCL10, CXCL12, CXCL14 and the immune microenvironment in HCC. The co-express gene of these chemokines can regulate immune and inflammatory reaction, as well as participate in signaling ways of chemokines, Toll-like receptors and tumor necrosis factor (TNF) [51]. Xu et al. investigated the action of group-2 innate lymphoid cells (ILC2s) in HCC. They discovered that ILC2s promoted the progress of HCC by stimulating the production of CXCL2 and CXCL8, which recruited neutrophils to create an immunosuppressive microenvironment, leading to the development of HCC [52]. According to these studies, it can be speculated CXCLs have markable potential to be biomarkers of HCC or to provide relevant treating strategy to HCC.

#### Ovarian cancer

Ovarian cancer (OC) is a kind of deadly carcinoma among gynecologic disease. There are accumulating researches associated with CXCL family in ovarian carcinoma. For instance, Park et al. concluded that CXCL1-CXCR2 axis contributed to the communication between epithelia and stroma, helping the growth of ovarian carcinoma via stimulating p38 [53]. After detecting and statistically analyzing, CXCL2 was validated that its overexpression has correlation with the physiological processes of epithelial ovarian cancer as well as poor prognosis [54]. CXCL10 was also proved to regulate tumor immune microenvironment of ovarian cancer, leading to strong immune cell infiltration. It can activate immune killing response and suppress angiogenesis, exerting anti-tumor function. Thus it was suggested to be a potential direction to improve OC-related immunotherapy [55, 56]. Moreover, researchers also found that the upregulation of CXCL14 facilitated OC-related tumor cell proliferation through STAT3 signaling pathway, while promoted epithelial-mesenchymal transition and tumor metastasis through Wnt/β-catenin pathway [57, 58]. All of these researches elucidated the significance of CXCLs for OC, supporting the relevant exploration of therapeutic value of CXC chemokines acting as biomarkers or targets.

#### Other cancers

In addition to the cancers mentioned above, CXC chemokines are also involved in the pathological mechanisms of prostate cancer, kidney renal clear cell carcinoma, melanoma, oral cancer, etc. For example, CXCL5 has autocrine and paracrine effects on malignant prostate cancer in prostate neoplasm cells and stroma which overexpress CXCL5, as well as participates in the regulation of tumor-related genes such as CXCR2, BAX, and ERK1/2 [59]. GNA13 (α subunit of a heterotrimeric G-protein), which can stimulate expression of CXCL5 via NF-κB pathway, becomes a novel therapeutic target due to the effect of this chemokine on prostate cancer [60]. Besides, CXCL5 secreted by renal clear cell cancer cell can form a positive feedback that induces the transformation from normal to tumor-related fibroblast to facilitate tumor growth [61]. In addition, Myakoshina et al. suggested that CXCL10 and CXCL12 promoted cell proliferation and angiogenesis in uveal melanoma respectively [62]. Zhang et al. indicated that level of CXCL1 had potential to be a biomarker of oral cancer due to its positive effect on the tumor-associated physiological behaviors such as proliferation and invasion, supporting the relevant researches for targeting treatment [63].

**Fig. 1:**
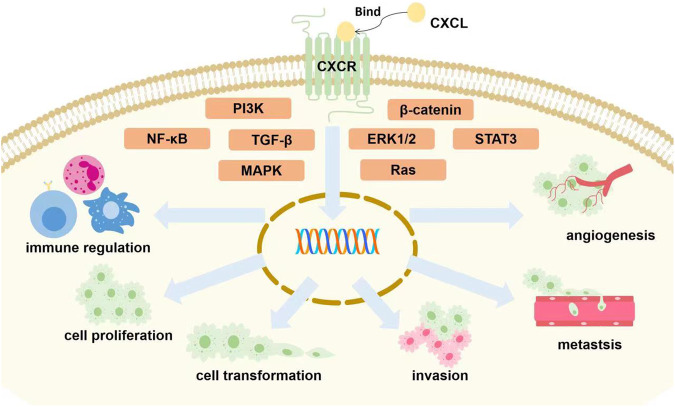
The roles of CXCL family in cancers. Majority of CXCL chemokines binding to corresponding receptors are involved in tumor growth via the activation of different signaling pathways (STAT3 [29, 105], NF-κB [31], Ras [7], MAPK [131], PI3K [47], TGF-β [7], β-catenin [57], ERK1/2 [47]). It eventually affects proliferation, invasion, migration and transformation of cancer cells, immune control, and tumor angiogenesis [24, 47, 48, 58, 63, 69].

In summary, CXCL family extensively takes part in immune reaction and biological processes of tumor, which contains angiogenesis, cellular transformation, tumor cell invasion, cancer metastasis to specific site and so on [19]. Apart from it, CXCL family is also usually used as biomarkers and therapeutic targets of various cancers, as well as used for prognostic and diagnostic, promoting the treatment of cancers. Therefore, CXCLs played an indispensable role in cancers. The main information of CXCLs and cancers is shown in Table 1 and Fig. 1.

**Table 1:** Receptors and related cancers of CXCL family.

CXCL	Alternative Name	Receptor	Related cancers	Type	Reference
CXCL1	Growth-related oncogene (GROα)	CXCR2	Lung cancer	In vivo	[20]
			Gastric cancer	In vivo	[27]
			Colorectal Cancer	In vivo and vitro	[31]
			Oral cancer	In vivo and vitro	[63]
			Breast cancer	In vivo	[37]
			Pancreatic cancer	In vivo and vitro	[43]
			Ovarian cancer	In vitro	[53]
CXCL2	GRO-β/macrophage inflammatory protein-2 (MIP-2)	CXCR2	Lung cancer	In vivo	[20]
			Gastric cancer	In vivo	[27]
			Colorectal cancer	In vivo and vitro	[32]
			Ovarian cancer	In vivo	[54]
			Liver cancer	In vivo and vitro	[48]
CXCL3	GRO-γ	CXCR2	Pancreatic cancer	In vivo and vitro	[43]
CXCL4	Platelet factor 4 (PF-4)	CXCR3	Gastric cancer	In vivo	[27]
			Breast cancer	Bioinformatics analysis	[40]
CXCL5	Epithelial-derived neutrophil-activating factor-78 (ENA-78)	CXCR1/2	Lung cancer	Bioinformatics analysis	[22]
			Gastric cancer	In vivo and vitro	[29]
			Pancreatic cancer	Biomatics analysis	[42]
			Liver cancer	In vivo and vitro	[47]
			Prostate cancer	In vivo and vitro	[59]
CXCL6	Granulocyte chemotactic protein-2 (GCP-2)	CXCR1/2	Lung cancer	In vivo and vitro	[132]
			Gastric cancer	In vivo	[133]
			Cervical cancer	In vivo and vitro	[134]
CXCL7	Neutrophil-activating polypeptide-2 (NAP-2)	CXCR2	Lung cancer	Bioinformatics analysis	[22]
			Gastric cancer	In vivo	[27]
CXCL8	Interleukin-8 (IL-8)	CXCR1/2	Lung cancer	In vivo	[20]
			Gastric cancer	In vivo	[27]
			Colorectal cancer	In vivo	[30]
			Breast cancer	In vivo	[37]
			Pancreatic cancer	In vivo	[44]
			Liver cancer	In vitro	[49]
CXCL9	Monokine induced by gamma interferon (MIG)	CXCR3	Gastric cancer	Bioinformatics analysis	[28]
			Breast cancer	Bioinformatics analysis	[40]
CXCL10	interferon ϒ-inducible protein 10 (IP-10)	CXCR3	Gastric cancer	Bioinformatics analysis	[28]
			Liver cancer	Bioinformatics analysis	[51]
			Ovarian cancer	Bioinformatics analysis	[56]
			MelanomaI	In vivo	[62]
CXCL11	Human interferon inducible T cell alpha chemokine (I-TAC)	CXCR3	Gastric cancer	Bioinformatics analysis	[28]
			Colorectal cancer	In vivo, vitro and bioinformatics analysis	[33]
CXCL12	Stromal cell derived factor 1 (SDF-1)	CXCR4/7	Lung cancer	In vivo	[25]
			Gastric cancer	In vivo	[27]
			Colorectal cancer	In vivo and vitro	[35]
			Breast cancer	Bioinformatics analysis	[40]
			Liver cancer	Bioinformatics analysis	[51]
			Melanoma	In vivo	[62]
CXCL13	B-lymphocyte chemoattractant(BLC)/B-cell attracting chemokine 1 (BCA-1)	CXCR3/5	Lung cancer	In vitro	[26]
			Gastric cancer	In vivo	[27]
			Colorectal cancer	In vivo	[30]
			Breast cancer	In vivo	[38]
CXCL14	Breast and kidney-expressed chemokine (BRAK)	CXCR4	Gastric cancer	In vivo	[27]
			Breast cancer	Bioinformatics analysis	[40]
			Liver cancer	Bioinformatics analysis	[51]
			Prostate cancer	In vitro	[135]
			Ovarian cancer	In vivo and vitro	[58]
CXCL16	Scavenger receptor that binds phosphatidylserine oxidized lipoprotein (SR-PSOX)	CXCR6	Gastric cancer	In vivo and vitro	[131]
			Colorectal cancer	In vivo and vitro	[136]
			Liver cancer	In vivo and vitro	[137]
			Breast cancer	In vitro	[138]
CXCL17	Dendritic cell and monocyte-like protein (DMC)/VEGF co-regulated chemokine 1 (VCC-1)	CXCR8	Gastric cancer	Bioinformatics analysis	[28]
			Colorectal cancer	In vivo	[139]
			Breast cancer	In vivo and vitro	[39]

### Inflammatory disease

#### Lung inflammation

CXCLs can cause diverse pneumonia via their role of chemotaxis. For instance, previous studies revealed that CXCL9 and CXCL10 may be implicated with deadly COVID-19 respiratory failure due to their participation in the recruitment of T cells and macrophages through a feedforward pathological signaling loop generated by TNFα and IFN-γ synergy in the inflamed lung [64]. Similarly, Fang et al. found that CXCL16 can recruit lymphocytes by chemotaxis via binding to CXCR6 to regulate immune system in the pathogenesis of Mycoplasma pneumoniae pneumonia [64].

COPD is a good example among all of CXCL-associated pulmonary inflammation. COPD is a chronic lung inflammatory disease which has the characteristic symptom of limited airways. It had been demonstrated that CXCL1 and CXCL8 were deeply associated with the pathogenesis of COPD. Previous studies have proved that CXCL8, which contributes to the migration of inflammatory cells to lung, is vital in COPD. It has been regarded as a biomarker of COPD severity [65]. In spite of it, one study indicated that lipopolysaccharide (LPS) and TNFα stimulated bronchial epithelial cells to produce CXCL1, and the LPS-induced process was involved in by NF-kB and MAPK p38. What’s more, this study concluded that CXCL1 was a better diagnostic marker than CXCL8 because therapeutic anti-inflammatory agents had less effects on CXCL1 production [66]. Besides, CXCL7 was also proved to promote the migration and adhesion of immunocytes to bronchial mucosa, causing the stronger airway inflammatory reaction in COPD [67]. Based on the important role of relevant CXCL family mentioned above in COPD, CXCL family can be used as feasible therapeutic targets.

#### Arthritis

CXCLs have close connection with different kinds of arthritis due to the role of CXCL about recruiting and migrating immune cells. For example, as to osteoarthritis, it was revealed by Caxaria et al. that CXCL6 stimulated inflammation via recruiting and activating immune cells and a therapeutic strategy to disrupt the capacity of CXCL6 to bind glycosaminoglycans for the aim of dispelling inflammatory reaction was proposed according to this discovery [68]. Apart from it, rheumatoid arthritis, a chronic autoimmune disease, also has strong relationship with CXCLs. Previous studies had speculated that extracellular vesicles derived from platelets may facilitate the activation of I-κB and NF-κB pathways through CXCL7/CXCR2 axis in order to promote the tumor-associated biological changes of fibroblast-like synoviocytes in rheumatoid arthritis [67, 69]. Other researches showed that CXCL10 was recommended as a disease activity marker of early rheumatoid arthritis, as well as serum CXCL13 level was closely related to rheumatoid arthritis and rheumatoid factor levels [70, 71]. In addition, the relevance between CXCL family (such as CXCL9, CXCL10, CXCL11 and CXCL12) and psoriatic arthritis has been explored by many studies [72, 73].

#### Liver inflammation

Pro-inflammatory CXC chemokines are extensively implicated in liver inflammation. CXCL1 and CXCL6 were proved by Jiang et al. that they were raised due to ethanol in the models of early alcoholic hepatitis, which showed the clinical value as the biomarkers of alcoholic liver disease [74]. Other studies have shown that CXCL2 and CXCL13 were closely correlated with liver inflammation because of their effects of recruiting inflammatory cells. Small heterodimer partner controlled the transcription of CXCL2, thereby controlling the recruitment of neutrophils [75]. It provided an alternative strategy to treat hepatitis. Similarly, dibutyl phthalate and benzopyrene gave rise to the imbalance of CXCL13, eventually resulting in liver inflammation and injury [76].

CXCL10 plays a dominant role in the pathogenesis of hepatic inflammation. For instance, CXCL8, CXCL9, CXCL10 and CXCL11, whose expression and serum concentration were increased after HBV infection, were thought to be hub genes and potential biomarkers of chronic hepatitis B. These chemokines produced a concentration gradient signal that assisted the recruitment of inflammatory cells such as neutrophils and T lymphocytes and promoted the occurrence of inflammatory reaction [77]. With regard as concanavalin A-induced hepatitis, the interaction between kupffer cells and infiltrated monocytes via CXCL10 takes effect [78]. Besides, hepatitis C virus and other immune-mediated hepatitis was participated in by CXCL10 according to previous studies, too [78, 79].

Nonalcoholic steatohepatitis (NASH) is closely related to CXC chemokines, too. CXCL family plays a crucial role in the inflammation development of NASH through recruitment and infiltration of immune cells including macorphages, neutrophiles, natural killer T cells and so on [80]. According to bioinformatics analysis, CXCL2 is one of the key genes associated with NASH [81]. CXCL5 expression elevated in NASH cells and can be inhibited via Livogrit to alleviate the progression of NASH [82]. CXCL10 also takes a crucial part in NASH. It was indicated to impair autophagic flux and autolysosome formation, thus leading to the inhibition of autophagic protein degrading and ubiquitinated protein accumulating and eventually causing the development of steatohepatitis [83].

#### Systemic lupus erythematosus

Systemic lupus erythematosus (SLE) is a common autoimmune disease. Xu et al. had demonstrated that the essential role of fisetin in treating SLE via regulating Th17 differentiation during lupus nephritis development and targeting the CXCL signaling pathway which decreased the expression of CXCL1, CXCL2 and CXCR2 [84]. Their study can show the importance of CXCL family in SLE. Besides, the correlation between CXCL10 and SLE were extensively studied. CXCL10 was found by Chorin et al. that it was significantly different in SLE compared to healthy controls. This chemokine was suggested to be a biomarker of disease activity and injury in SLE [85]. Another group evaluated the correlation between gene expression level of CXCL10 and SLE disease activity via Fold Change method. This group concluded that different SLE activity had different levels of CXCL10 gene expression, and the gene expression of severe activity group was remarkably elevated [86]. Thus, CXCL10 is not only a possible biomarker of severe lupus activity, but also a feasible target in SLE therapeutic strategy. For instance, one study mentioned that using baricitinib could downregulate key cytokines like CXCL10 in order to treat SLE [87].

#### Kidney inflammation

Similar to liver inflammatory diseases, kidney inflammatory diseases were correlated with proinflammatory chemokines of CXCL family. Many authors showed that the activation of renal vascular endothelium promoted the production of immunological CXC chemokine molecules. Take CXCL8 for a example, it was proved to recruit the neutrophils, assisting the activation of inflammatory pathways in the ischemic kidney and culminating the acute kidney injury [88]. As another proinflammatory gene, CXCL5 also makes the similar effect on renal inflammation and impairment of kidney function as CXCL8 [89].

Lupus nephritis (LN), a famous kidney inflammation, is one of the main clinical symptoms of systemic lupus erythematosus (SLE). There are many relevant researches explaining the relationship between LN and CXCLs. Khan et al. had explored the ability of arachidonic acid epoxyeicosatrienoic acid (EET) analog to protect kidney and prevent its injury in a SLE mouse model. After a series of analysis they found that SLE mice exhibited remarkable renal chemotaxis and increased renal mRNA expression of CXC chemokines such as CXCL13 and CXCL16 compared to Non SLE mice, whereas EET analog therapy altered this situation so that the mRNA expression levels of CXC chemokines and its receptors as well as immunocytes infiltration associated with kidney was decreased [90]. Other studies also clearly suggested the critical role of CXCL16 in the pathogenesis of LN and their clinical values as reliable indicator [91, 92]. Hassan et al. proposed that CXCL16 was an essential mediator in renal inflammation diseases like LN. They found that serum CXCL16 level was markedly higher in juvenile patients with SLE than healthy controls, as well as associated with SLE-related disease indexes such as disease activity and severity of relevant nephritis, ultimately speculated CXCL16 may exert biological functions in LN [91]. In addition, accumulated evidences also indicated that CXCL10 also participated in LN. It not only is responsible for the attraction to T cells and the renal inflammation, but also takes part in the pathogenesis of LN [93].

#### Organ injury and fibrosis

Acute kidney injury (AKI) is a serious health problem caused by sepsis, ischemia, nephrotoxic insult and so on. It often results in chronic kidney disease and renal failure. One previous research had studied the role of the interaction between CXCR3 and its corresponding CXC ligands in renal ischemic injury through inducing renal ischemia in mouse model. Finally, they discovered that it played an essential role in the recruitment of Th1 cells to kidney and mediating the renal ischemia-reperfusion injury [94]. Currently, researchers identified again that infiltrating immune cells can influence the process of AKI by the signaling pathways of CXC chemokines (such as CXCL1/CXCR2 axis) and so on [95]. In addition, Medeiros et al. analyzed COVID-19 patients evolving with AKI. It was found that AKI patients allowed higher CXCL8 and CXCL10, showing a predictive value diagnosing the occurrence of AKI [96]. CXCL12-CXCR4/7 also joined in repairment of renal ischemia-reperfusion injury via mediating the migration of mesenchymal stem cells [97, 98]. Thus it can be proposed the possibility of considering CXCLs as a promising therapeutic target for AKI.

Acute lung injury is a severe imbalanced lung inflammatory reaction characterized by loss of alveolar epithelial and capillary barrier function [67]. According to the role of CXCL family, it was discovered that palmatine can reduce the expression of CXCL1 and CXCL2 to lower the infiltration of inflammatory cells and relieve lung injury induced by LPS [99]. What’s more, Wang et al. explained the mechanism of resolvin D1 attenuating LPS-induced acute lung injury. CXCL12-CXCR4 pathway was involved in neutrophil accumulation and retention in the inflammatory site. Resolvin D1 regulated this signaling pathway to avoid excessive neutrophils accumulation and prevent from forming acute lung injury [100].

Acute liver injury is closely related with CXCL1, CXCL2 and other chemokines. Acetaminophen-induced liver injury has obviously more expression of CXCL1, activating the recruitment of neutrophils [101]. Like CXCL1, CXCL2 can also recruit neutrophils to cause liver injury due to small heterodimer partner deficiency [75].

Due to the sustained inflammatory injury, organ injury usually leads to fibrotic diseases. Among different fibrosis, liver fibrosis and lung fibrosis are the most common in researches concerned with the effect and application of CXCLs. Zhang et al. had investigated the pathogenic mechanism of liver fibrosis in Schistosoma japonicum-infected mice. At last they preliminarily explored the immunological network of liver fibrosis, which included the role of CXCL16-CXCR6 pathway about recruiting Th2 cells [102]. Considering CXCL family as targets, Hermert et al. provided the evidence that the nucleic acid binding protein YB-1 can control hepatic fibrosis through regulating CXCL1 expression [103]. As to the correlation lung fibrosis with CXCL4, Bon et al. observed that CXCL4 level was implicated with lung fibrosis. It was concluded that CXCL4 performed different functions when in vitro and in vivo. In the former, CXCL4 reduced FLI1(transcription factor) expression, activated endothelial-cell markers, and strengthened responses of toll-like receptors, while in the latter, it promoted the entry of inflammatory cells and the changes of skin transcriptome [104]. Other kinds of organ fibrosis are also closely related to CXCLs. For instance, Liu et al. demonstrated that CXCL13 overexpression was responsible for promoting prostate inflammatory reaction and fibrosis of WPMY-1 cell (human prostate cell lines) via STAT3 signaling pathway [105].

#### Ovarian inflammation

Endometriosis is an in flammatroy female diseases. CXCLs can affect relative inflammation process in endometriosis. For example, one study showed that CXCL16 expression was found to increase in endometriosis compared to control group. CXCL16-CXCR6 axis can be activated by TNF-α, leading to migration and invasion of ectopic endometric stem cells. That meant CXCL16 was associated with progression of endometriosis [106]. Zhang et al. discovered that CXCL1, CXCL2 and CXCL5 were expressed at endometric lesion, which contributed to the recruitment of myeloid-derived suppressor cells to treat endometriosis by suppressing immue responses and promoting angiogenesis [107]. CXCLs also have potentials to cure for this disease. CXCL12 was confirmed to enhance pregnancy rates in endometriosis mouse model, considered as a potential agent for therapy of endometriosis [108]. Polycytics ovary syndrome (PCOS) usually has inflammatory response. One previous study has proverd that the differential expression of CXCL family mRNA may participate in inflammatory reations in mouse model [109].

#### Other inflammatory diseases

Chen et al. studied the role of CXCL13 in spinal cord ischemia-reperfusion injury rat model. They confirmed that CXCL13 siRNA reduced proinflammatory cytokines. CXCL13 was potentially involved in the development of spinal cord ischemia-reperfusion injury through ERK pathway [110]. Besides, Lin et al. proved that CXCL12 contributed to remyelination in central nervous system and alleviation of experimental autoimmune encephalomyelitis in vivo study [111].

Other inflammatory diseases related with CXCL family contain ovarian related inflammation, inflammatory bowel disease, organ transplants, vascular diseases and so on [67, 112–114].

**Fig. 2:**
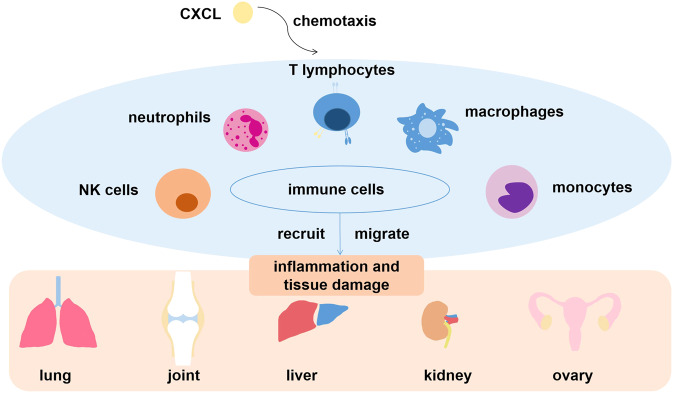
The role of CXCL family in inflammatory diseases. CXC chemokines assist the recruition and migration of diverse immune cells to different organs or tissues, eventually leading to inflammatory reactions.

In summary, CXCLs have the important ability to recruit and migrate various immunological cells like neutrophils and T cells in order to induce inflammatory reaction, leading to different kinds of inflammatory diseases mentioned above and tissue damage (Fig. 2). According this characteristics, CXCLs are usually applied in inflammatory diseases as diagnostic indicators or therapeutic targets, which is similar to their usage in cancers.

### Metabolic disease

#### Diabetes

Type 1 diabetes (T1D) results from the T cell-mediated damage of pancreatic β cells which provide insulin. CXCL10-CXCR3 axis was thought to be connected with T1D by many authors. They have explained that pancreatic β cells produced CXCL10, which attracted autoreactive T cells and macrophages to the pancreatic islets by binding to CXCR3, resulting in the destruction of β cells. Based on the pivotal role of CXCL10-CXCR3 pathway in T1D, blocking this pathway may be an extra strategy for T1D treatment [115–117]. According to the study of Li et al. related with human mesenchymal stem cells (hMSCs) in high-glucose environment, it can been seen that hMSCs can increase islet β cells’ activity via p-AKT pathway because of the chemotaxis of CXCL13 [118]. Different from T1D, type 2 diabetes (T2D) is mainly the result of insulin resistance (IR). Moreno et al. had studied many obese patients and ultimately found that CXCL10 and CXCL11 were associated with IR in obesity [119]. Another study showed that in T2D the migration in responses to CXCL12 was declined by diabetic fibrocytes while the migratory of fibrocytes contributed to wound healing and chronic ulcer caused by diabetes [120]. Apart from the two types of diabetes mentioned above, there is also gestational diabetes mellitus (GDM) interrelated to CXC chemokines. Liu et al. established that the elevated expression of CXCL1, CXCL8, CXCL9 and CXCL12 as well as the declined expression of CXCL10 were associated with GDM. Thus, it was necessary to regard these chemokines as possible biomarkers and targets [121]. Besides, the abnormal expression of CXCL16 was mentioned to influence GDM [122].

Furthermore, diabetes also develops many derivative diseases. For instance, CXCL1 and other chemokines were increased in diabetic ketoacidosis mice model. The chemokines can be reduced by triarylmethane-34 and minocycline, relieving neuroinflammation [123]. Diabetes can provoke diabetic foot ulcers. Researchers had shown that hyperbaric oxygen therapy stimulated angiogenesis via the increased CXCL8 and CXCL10 level, helping the healing of chronic wounds [124].

#### Polycystic ovary syndrome

Polycystic ovary syndrome (PCOS) is a female endocrine disorder and a reproductive disease that affects fertility and life health of about 10% women around the world [125]. CXCL13-CXCR5 axis exerts the regulatory function in inflammatory reactions. According to PCOS mouse model trials, one study showed that CXCL13 and CXCR5 expressions were increased in the tissue samples of PCOS mice. It concluded that CXCL13 and CXCR5 might take part in the pathgenesis of PCOS [126]. Qi et al. evaluated the role of CXCL14 in PCOS and discovered that CXCL14 upregulated steroidogenic acute regulatory expression in human-luteinized granulosa cells, contributing to steroidogenesis failure in granulosa cells of PCOS patients [127]. CXCL14 can protect girls with PCOS from insulin resistance. Another study indicated that low level of CXCL14 in girls with PCOS would return to normal by using insulin sensitization [128].

#### Other metabolic diseases

Meng et al. had discovered that CXCL-13 was involved in the increased accumulation of lipid droplets and higher expression of FAS and SREBP1, while it inhibited AMPK pathway and stimulated the PKC and P38 pathways to regulate abnormal lipid metabolic disorder. They eventually demonstrated that the upregulated CXCL13-related pathway participated in the process of hyperuricemia inducing lipid disturbances [129]. CXCL9 participates in metabolic-associated fatty liver disease via facilitating the imbalance of Treg/Th17 cells with interferon-gamma, providing another new direction to treat this disease [130].

In fact, researches associated with the relationship between CXCL family and metabolic diseases are increasing nowadays, since the therapeutic potential of CXCL in metabolic disease are showing to world [80]. CXCL family may perform functions in the process of metabolic diseases via recruitment of immune cells and regulation of inflammation.

## Conclusion

The relevant researches of CXCL family are concentrated in tumors, inflammatory diseases and metabolic diseases. In the pathogenic mechanisms of diverse diseases, CXC chemokines usually play an essential role in regulating inflammation and immune responses through the migration of variety of leukocytes including neutrophils, monocytes, macrophages, etc. It affects tumor cells proliferation, invasion, metastasis, and angiogenesis via a variety of signaling pathways like STAT3 and NF-κB, thus accelerating the development of cancers. These features give CXC chemokines a special position in cancers, inflammatory diseases and so on. Although the importance of CXCLs in these diseases has been understood, the concrete mechanism of action for CXCLs in pathogenesis and progression of relevant diseases is still not completely clarified, and their relevant therapeutic methods and clinical attempts is still not extensively explored. That is one of things what researchers should investigate it in the future. However, it can be known that the abnormal expression of CXCL family in cancers and inflammation can serve as potential biomarkers, targets, indicators and so on, which contributes to the diagnosis, therapy and prognosis of certain diseases. CXCL family is a promising direction in future researches for treatment of carcinoma, inflammatory diseases, metabolic diseases, etc. The further research about pre-clinical and clinical applications based on CXCL family should be carried out in the future.

## Methods

Based on PubMed, Web of Science and other databases, this paper retrieved relevant articles from January 2015 to December 2022, with the key words of “CXCL” or “CXC chemokines” or “CXCL and cancer” or “CXCL and inflammatory disease” or “CXCL and metabolic disease” or “CXCL and liver” or “CXCL and lung”, etc. Ultimately the articles related to CXCL family and its effects on relevant diseases were screened out, containing reviews, clinical trials and animal studies.
